# Maternal and Neonatal Complications After Natural vs. Hormone Replacement Therapy Cycle Regimen for Frozen Single Blastocyst Transfer

**DOI:** 10.3389/fmed.2020.00338

**Published:** 2020-08-28

**Authors:** Jia Lin, Junzhao Zhao, Guimin Hao, Jichun Tan, Ye Pan, Ze Wang, Qi Jiang, Ning Xu, Yuhua Shi

**Affiliations:** ^1^Center for Reproductive Medicine, Cheeloo College of Medicine, Shandong University, Jinan, China; ^2^National Research Center for Assisted Reproductive Technology and Reproductive Genetics, Shandong University, Jinan, China; ^3^Key Laboratory of Reproductive Endocrinology of Ministry of Education, Shandong University, Jinan, China; ^4^Shandong Provincial Clinical Medicine Research Center for Reproductive Health, Shandong University, Jinan, China; ^5^Reproductive Medicine Center, The First Affiliated Hospital of Wenzhou Medical University, Wenzhou, China; ^6^Department of Obstetrics and Gynecology, Reproductive Medical Center, The Second Affiliated Hospital of Wenzhou Medical University, Wenzhou, China; ^7^Department of Reproductive Medicine, The Second Hospital of Hebei Medical University, Shijiazhuang, China; ^8^Department of Obstetrics and Gynecology, Reproductive Medicine Center, Shengjing Hospital of China Medical University, Shenyang, China; ^9^School of Rehabilitation, Shandong University of Traditional Chinese Medicine, Jinan, China

**Keywords:** biomedical miscarriage, frozen embryo transfer, natural cycle, hormone replacement therapy cycle, single embryo transfer

## Abstract

**Objective:** To evaluate the maternal and neonatal complications after frozen-thawed blastocyst transfer cycles utilizing different endometrial preparation regimens.

**Design:** This is a retrospective cohort study and a secondary analysis of a multicenter, randomized, controlled trial comparing live birth rate after fresh vs. frozen single blastocyst transfer (Frefro-blastocyst).

**Setting:** Reproductive medicine centers.

**Patient(s):** A total of 800 women with regular menstrual cycles undergoing their first cycle of *in-vitro* fertilization after frozen-thawed single blastocyst transfer.

**Intervention(s):** Endometrium preparation was performed with a natural cycle regimen or hormone replacement therapy (HRT) cycle regimen, at the discretion of local investigators. All pregnancies were followed up until delivery.

**Main Outcome Measure(s):** Maternal and neonatal complications.

**Result(s):** 513 infertile patients who underwent natural cycles regimen and 287 who underwent HRT cycles regimen were analyzed. The incidences of maternal and neonatal complications were comparable between the natural cycle and HRT cycle regimen. Regarding the risk of gestational diabetes, gestational hypertension, pre-eclampsia, preterm delivery, small for gestational age and large for gestational age, the HRT cycle was still not a significant risk factor after adjusting for potential confounders. The natural cycle regimen yielded an insignificant higher total live birth rate [59.45 vs. 50.17%, *P* = 0.001, adjusted odds ratio (AOR) 1.366, 95% confidence interval (CI) 0.975–1.913], clinical pregnancy rate (68.23 vs. 58.89%, *P* = 0.008, AOR 1.406, 95% CI 0.992–1.991) and ongoing pregnancy rate (62.18 vs. 52.61%, *P* = 0.008, AOR 1.387, 95% CI 0.988–1.948) than did the HRT cycle regimen. However, compared to natural cycles, HRT cycles were associated with a significantly higher risk of biochemical miscarriage (6.86 vs. 18.18%, *P* < 0.001, AOR 0.328, 95% CI, 0.176–0.611).

**Conclusion(s):** The incidence of maternal and neonatal complications in natural cycle and HRT cycle regimens after frozen single blastocyst transfer were comparable. Frozen-thawed single blastocyst transfer in a natural cycle was associated with lower biomedical miscarriage than the use of the HRT cycle.

**Clinical Trial Registration Number:** Frefro-blastocyst was registered at Chinese Clinical Trial Registry, ChiCTR-IOR-14005405.

## Introduction

The goal of assisted reproductive technology (ART) is to achieve a live, healthy, full-term singleton baby. Single embryo transfer (SET) is the most efficient approach to reduce the risk of multiple pregnancies (1). With the development in cryopreservation technology, especially the introduction of vitrification, the application of frozen embryo transfer (FET) has become increasingly popular (2). Studies have shown that frozen embryo transfer by avoiding a supra-physiological environment for embryo implantation after ovarian stimulation increased pregnancy rate (3, 4). By prolonging the *in vitro* culture, only embryos of excellent quality with the highest potential for implantation will reach blastocyst stage selection. Blastocyst transfer has resulted in significantly higher rates of pregnancy and delivery than cleavage-stage embryo transfer (5).

Our previous trial suggested that frozen single blastocyst transfer was better for achieving singleton live birth than fresh single blastocyst transfer in women with good prognosis (6). However, frozen single blastocyst transfer was associated with a higher risk of pre-eclampsia (6). Further, higher rates of hypertensive disorders and placenta accreta in pregnancy were noted after frozen embryo transfer (7). FET singletons may be at an increased risk of being born large for gestation age (LGA) (7, 8). Whether these differences are due to the protocol used in frozen cycles remains unknown.

A crucial aspect of FET cycles is the preparation of the endometrium to receive the transferred embryo. The most commonly used endometrial preparation methods for FETs include hormone replacement therapy (HRT) cycle and natural cycle (NC). Many retrospective analyses were performed of frozen blastocyst transfers, and different conclusions were drawn about the implantation, pregnancy, and live birth rate (9–12). The results of meta-analysis and systematic review show that no sufficient evidence has been found to support the superiority of one method over the other (2, 13). While few studies have investigated the neonatal and maternal outcomes comparing these strategies, compared to natural cycle protocols, higher rates of hypertensive disorders in pregnancy (14, 15), postpartum hemorrhage (14, 16), post term birth (14), macrosomia (14), and cesarean section (15) were detected in the HRT cycle. It seemed that the HRT cycles of FET have a negative effect on obstetric outcome.

In this study, a secondary analysis was performed to see whether the method of endometrial preparation for transfer of vitrified blastocysts was associated with obstetric complications outcomes.

## Materials and Methods

### Study Population

The Frefro-blastocyst study was conducted during August 1, 2016, to June 3, 2017 in 21 academic fertility centers in China. The original study was approved by the ethics committees of all study sites and was registered at Chinese Clinical Trial Registry (number ChiCTR-IOR-14005405) (6). The design and main outcomes of this trial have been previously reported in detail (17). Briefly, 1,650 women with regular menstrual cycles undergoing their first cycle of *in vitro* fertilization were enrolled, and eligible women were randomly assigned to either fresh or frozen single blastocyst transfer. For those assigned to frozen blastocyst transfer, all blastocysts were cryopreserved, and a delayed frozen-thawed single blastocyst transfer was done. Considering the condition of switched groups in the randomized clinical trial (RCT), 724 patients adhered to frozen protocol and 87 women assigned to the fresh embryo transfer group who actually had single frozen blastocyst transfer were included. However, only natural cycle and HRT cycle regimens for endometrium preparation of FET were analyzed.

### Study Procedure

After ovarian stimulation with a gonadotropin-releasing hormone antagonist protocol, women who obtain four or more than four embryos on day three of the embryo culture were randomized into two groups: fresh single blastocyst transfer group and frozen single blastocyst transfer group. The selection of the single blastocyst gave priority to the score of the inner cell mass, and the score of trophectoderm was also considered. The rank of blastocyst grade from top to good was AA, AB, BA, BB, AC, and BC. If two or more blastocysts were of equal grade, their early scores at cleavage stage were referred for the selection of the single blastocyst. Supernumerary embryos were frozen on day 5 or 6 according to embryo development. On day 5 or 6 of embryo culture, women who were assigned to the frozen blastocyst transfer group had their blastocysts vitrified and a deferred frozen blastocyst transfer. The selection of the frozen blastocyst for thawing was based on the blastocyst grade before freezing.

### Endometrial Preparation Protocols

At least 4 weeks after blastocysts vitrified, endometrium preparation was performed with a natural cycle regimen or HRT cycle regimen. At the discretion of local investigators, this assignment was not randomized. For the natural cycle regimen, when it was detected that the dominant follicle and the endometrial thickness reached 7 mm or more, local investigators decided whether to use human chorionic gonadotropin (hCG) for ovulation triggering according to their clinical routine. Ovulation was determined by ultrasound monitoring and a single frozen-thawed blastocyst, either day 5 or day 6, was transferred on the 5th day after ovulation. Details of embryo transfer procedure were shown in a previous study (6). Luteal phase support was started after the ovulation day with oral dydrogesterone 10 mg three times daily. If the patient was pregnant, luteal phase support was continued until 10 weeks' gestation. For the HRT cycle regimen, the endometrium prepared with oral estradiol valerate (Progynova, Delpharm Lille, Lys-Lez-Lannoy, France) at a dose of 4–8 mg daily was started on days 1–3 of the menstrual cycle. Vaginal progesterone gel (Crinone, Merck Serono) 90 mg/day and oral dydrogesterone 10 mg twice daily were added when the endometrial thickness reached 7 mm or more. A single frozen-thawed blastocyst was transferred on the 5th day after progesterone initiation. Estradiol valerate at the dose for endometrium preparation was continued until the day of the serum hCG test, 2 weeks after embryo transfer. If pregnancy was achieved, estradiol valerate stopped gradually at 8–9 weeks of gestation; vaginal progesterone gel and oral dydrogesterone was continued until 10 weeks of gestation.

### Outcome Measures

The study outcomes included clinical pregnancy, ongoing pregnancy, pregnancy loss, live birth, ectopic pregnancy, perinatal complications and neonatal complication gestational, e.g., gestational diabetes mellitus (GDM), pregnancy-induced hypertension (PIH), pre-eclampsia, gestational age at birth, preterm birth, small for gestational age (SGA), large for gestational age (LGA), and neonatal hospitalization for more than 3 days. Preterm birth was defined as delivery before 37 complete weeks of gestation. The outcomes SGA and LGA were, respectively, defined according to birth weight for the 10th and 90th percentile of gender-specific birth weight reference for Chinese (18).

### Statistical Analysis

Continuous data were expressed as mean (SD) and compared by the Student's *t*-test. Categorical data were represented as frequency and percentage; differences in these variables between the treatment groups were assessed by χ^2^ analysis, with Fisher's exact test for expected frequencies less than five. A *P* < 0.05 was considered statistically significant. Crude odds ratios (OR) with 95% confidence intervals (CI) for each primary outcome were calculated. Logistic regression analysis was used to compare adjusted odds ratios (AOR) and 95% CI for the effect of natural cycle vs. HRT cycle regimen on obstetric and perinatal complications. Goodness of fit for logistic regression models were calculated by the Hosmer-Lemeshow (HL) test, if the *P* > 0.05, the model passed the test. All analyses were performed with the use of SPSS software 21.0.

## Results

**Figure 1:**
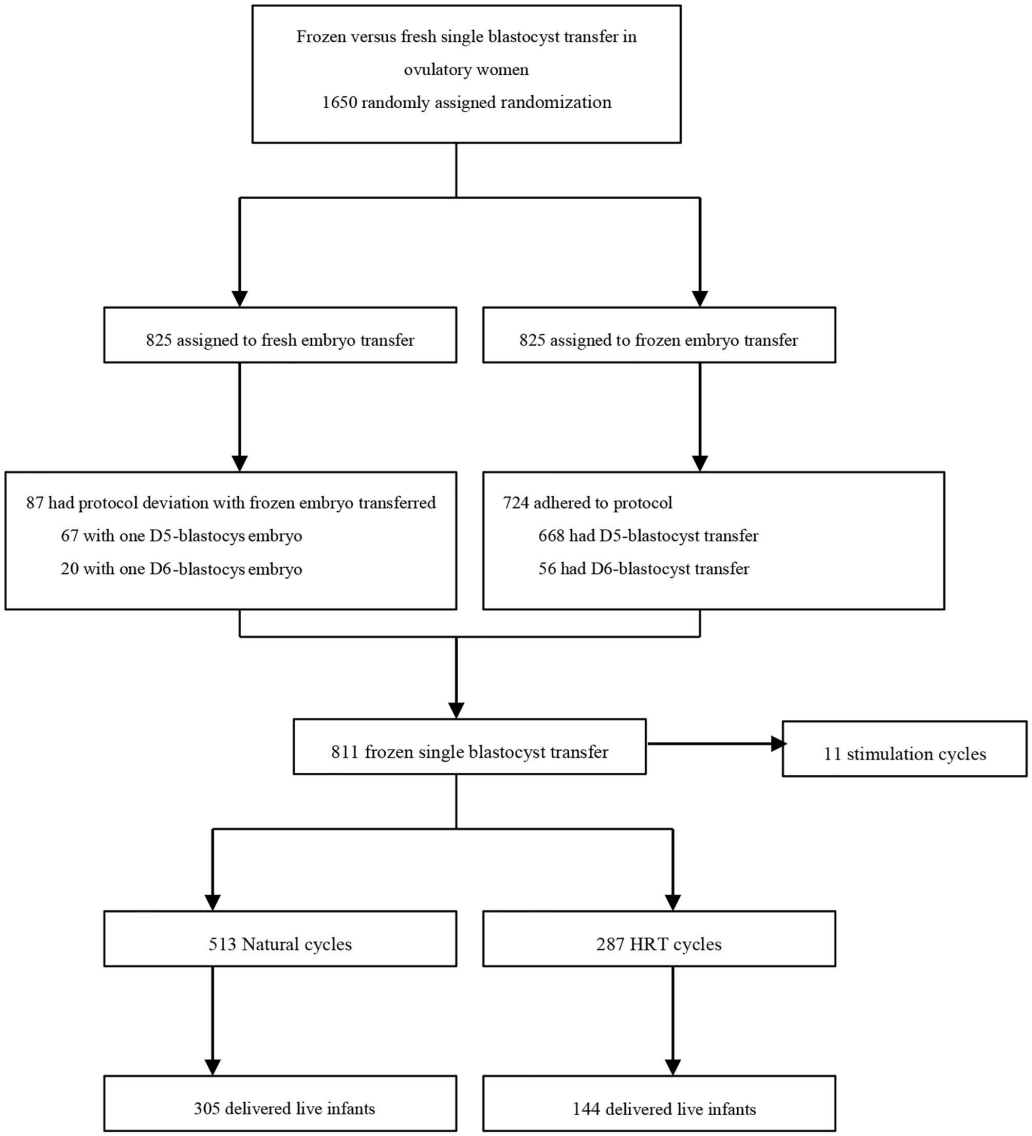
Study flow diagram.

Seven hundred and twenty-four women who were assigned to the frozen embryo transfer group actually adhered to the protocol, while 87 women assigned to the fresh embryo transfer group actually had a frozen embryo transfer. Of these, 11 women were excluded due to a stimulation cycle regimen for endometrial preparation of FET. 513 infertile patients who underwent natural cycles regimen and 287 who underwent HRT cycles regimen were analyzed (Figure 1).

Baseline characteristics are listed in Table 1. Maternal age, body mass index, duration of infertility, proportion of previous conception, the proportion of IVF indications, antral follicle count (AFC), baseline follicle stimulating hormone (FSH), baseline luteinizing hormone (LH), oestradiol (E_2_), days of ovarian stimulation, endometrial thickness on day of trigger, number of oocytes, fertilization method and incidence of ovarian hyper-stimulation syndrome (OHSS) were comparable between the natural and HRT cycles. However, in the natural cycles compared to the HRT cycles, total testosterone level, total gonadotropin dose, E_2_ and progesterone level on the day of trigger were significantly lower (*P* < 0.001, *P* = 0.02, *P* = 0.006, respectively), the number of 2PN embryos, cleavage embryos and day-3 suitable for transferred embryos were less (*P* = 0.015, 0.032, 0.015, respectively), and the endometrial thickness was thinner (*P* < 0.001).

**Table 1:** Baseline characteristics.

**Characteristics**	**Natural cycles (*n* = 513)**	**HRT cycles (*n* = 287)**	***P*-value**
Age (years)	28.74 ± 2.89	29.08 ± 3.01	0.117
Body-mass index (kg/m)	22.32 ± 3.07	22.52 ± 3.30	0.379
Duration of infertility (years)	3.26 ± 2.11	3.29 ± 2.35	0.846
Previous conception *n* (%)	219/513 (42.69)	133/287 (46.34)	0.318
Indications for IVF			0.364
Tubal factor	276/513 (53.80)	156/287 (54.36)	
Male factor	113/513 (22.03)	64/287 (22.30)	
Unexplained infertility	20/513 (3.90)	18/287 (6.27)	
Combined factors	104/513 (20.27)	49/287 (17.07)	
Antral follicle count in both ovaries	16.46 ± 5.46	16.87 ± 5.99	0.339
Baseline sex hormone			
FSH (IU/L)	6.34 ± 1.50	6.32 ± 1.46	0.812
LH (IU/L)	4.78 ± 1.93	4.83 ± 2.21	0.717
Oestradiol (pg/mL)	37.07 ± 14.60	37.94 ± 15.64	0.430
Total testosterone (ng/dL)	28.46 ± 14.03	35.96 ± 17.82	<0.001
Fresh cycle variables			
Days of ovarian stimulation	9.28 ± 1.40	9.49 ± 1.68	0.056
Total gonadotropin dose (IU)	1559.32 ± 479.52	1634.78 ± 500.26	0.036
Oestradial level on HCG trigger day (pmol/L)	3431.55 ± 1810.08	3758.37 ± 1801.59	0.020
Progesterone level on HCG trigger day (nmol/L)	1.14 ± 0.52	1.26 ± 0.58	0.006
Endometrial thickness on HCG trigger day (cm)	1.07 ± 0.20	1.06 ± 0.20	0.512
No. of oocytes retrieved	14.29 ± 5.74	14.69 ± 5.68	0.339
No. of 2PN embryos	9.43 ± 3.97	10.17 ± 4.53	0.015
No. of cleavage embryos	9.29 ± 3.96	9.94 ± 4.36	0.032
Fertilization method			0.333
IVF *n* (%)	359/513 (69.98)	210/287 (73.17)	
ICSI *n* (%)	127/513 (24.76)	68/287 (23.69)	
IVF +ICSI *n* (%)	27/513 (5.26)	9/287 (3.14)	
No. of day-3 embryos suitable for transfer	6.96 ± 2.99	7.59 ± 3.69	0.015
OHSS *n* (%)	3/513 (0.58)	4/287 (1.39)	0.258
FET cycle variables			
Endometrial thickness before transfer (cm)	1.00 ± 0.17	0.94 ± 0.16	<0.001
Timing of embryo transfer—no./total no. (%)			0.853
Day 5	465/513 (90.64)	259/287 (90.24)	
Day 6	48/513 (9.36)	28/287 (9.76)	
No. of remaining frozen blastocysts	4.19 ± 2.90	4.27 ± 3.00	0.720

The pregnancy outcomes after FET are shown in Table 2. The natural cycle regimen yielded a higher total live birth rate including singleton and twin than the HRT cycle regimen (59.45 vs. 50.17%, *P* = 0.011). Singleton live birth per woman was also higher in natural cycles (57.50 vs. 48.78%, *P* = 0.017), whereas twin live birth per woman was similar between the two protocols (*P* = 0.565). Clinical pregnancy (68.23 vs. 58.89%, *P* = 0.008) and ongoing pregnancy (62.18 vs. 52.61%, *P* = 0.008) were significantly higher in the natural cycles. Furthermore, the rate of biochemical miscarriage was significantly lower in the natural cycles (6.86 vs. 18.18%, *P* < 0.001). No significant differences were observed in terms of birth weight, gestational weeks, biochemical pregnancy and clinical pregnancy loss rate.

**Table 2:** Livebirth, birthweight, pregnancy, and pregnancy loss.

	**Natural cycles (*n* = 513)**	**HRT cycles (*n* = 287)**	***P*-value**
Total live birth per woman	305/513 (59.45)	144/287 (50.17)	0.011
Singleton live birth per woman	295/513 (57.50)	140/287 (48.78)	0.017
Twin live birth per woman	10/513 (1.95)	4/287 (1.39)	0.565
Birth weight			
Singleton (g)	3395 ± 476.14	3417.39 ± 480.02	0.654
Monozygotic Twin (g)	2424.50 ± 501.58	2365.00 ± 525.03	0.782
Gestational weeks (week)	38.9 ± 1.66	39.04 ± 1.74	0.464
Pregnancy			
Biochemical pregnancy—no. (%)	379/513 (73.88)	209/287 (72.82)	0.745
Clinical pregnancy—no. (%)	350/513 (68.23)	169/287 (58.89)	0.008
Ongoing pregnancy—no. (%)	319/513 (62.18)	151/287 (52.61)	0.008
Pregnancy loss-no./total no. (%)			
Total pregnancy loss among biochemical pregnancies	67/379 (17.68)	62/209 (29.67)	0.001
Biochemical miscarriage	26/379 (6.86)	38/209 (18.18)	<0.001
Clinical pregnancy loss	41/350 (11.71)	24/169 (14.20)	0.422
First trimester pregnancy loss	31/350 (8.86)	18/169 (10.65)	0.513
Second trimester pregnancy loss	10/350 (2.86)	6/169 (3.55)	0.669

Maternal and neonatal complications stratified by FET method are shown in Table 3. There were no significant differences in the incidence of ectopic pregnancy, GDM, PIH, pre-eclampsia, preterm rupture of membrane, preterm delivery and post-partum hemorrhage between the two groups. In addition, no significant between-group difference was found in the risks of small for SGA, LGA, neonatal hospitalization for more than 3 days, neonatal infection among live newborn, nor birth defect.

**Table 3:** Maternal and neonatal complications.

	**Natural cycles (*n* = 513)**	**HRT cycles (*n* = 287)**	***P*-value**
Maternal complications			
Ectopic pregnancy	3/350 (0.79)	2/169 (0.96)	0.663
Gestational diabetes	40/350 (11.43)	19/169 (11.24)	0.950
Gestational hypertension	8/350 (2.29)	5/169 (2.96)	0.765
Pre-eclampsia	9/350 (2.57)	6/169 (3.55)	0.580
Placenta previa	6/350 (1.71)	0	0.184
Preterm rupture of membrane	36/350 (10.29)	18/169 (10.65)	0.898
Preterm delivery	22/350 (6.29)	10/169 (5.92)	0.870
Post-partum hemorrhage	3/308 (0.97)	5/144 (3.47)	0.117
Neonatal complications			
Small for gestational age	16/310 (5.16)	10/144 (6.94)	0.447
Large for gestational age	58/310 (18.71)	27/144 (18.75)	0.992
Neonatal hospitalization >3 days	39/303 (12.87)	13/142 (9.15)	0.255
Neonatal jaundice among live newborns	55/303 (18.15)	26/142 (18.31)	0.968
Neonatal infection among live newborns	11/303 (3.63)	4/142 (2.82)	0.783
Birth defect	10/318 (3.14)	5/148 (3.38)	1.000

*Values are number (percentage)*.

Regarding the risk of gestational diabetes, gestational hypertension, pre-eclampsia, preterm delivery, SGA and LGA, the HRT cycle was still not a significant risk factor after adjusting the results for potential confounders. A multiple logistic regression analysis adjusted for potential confounders showed that natural cycle FET (vs. HRT cycle-FET) was a higher risk factor for live birth rate (AOR 1.411; 95% CI 1.011–1.970), clinical pregnancy rate (AOR 1.444, 95% CI 1.022–2.041), ongoing pregnancy rate (AOR 1.443, 95% CI 1.031–2.020) and a significant lower risk factor for biochemical miscarriage (AOR 0.323; 95% CI 0.174–0.599) in Model 1, which did not pass the HL test (Table 4). When adding age and FSH to adjust in Model 2, only the rate of biochemical miscarriage (AOR 0.328; 95% CI 0.176–0.611) was still significantly lower in natural cycles than in HRT cycles (Table 4).

**Table 4:** Logistic regression for the effect of natural cycle vs. HRT cycle regimen on obstetric complication.

			**Model 1**		**Model 2**	
	**Unadjusted OR (95% CI)**	***P*-value**	**Adjusted OR (95% CI)**	***P* value**	**Adjusted OR (95% CI)**	***P*-value**
Total live birth	1.456 (1.089–1.948)	0.011	1.411 (1.011–1.970)	0.043	1.366 (0.975–1.913)	0.070
Clinical pregnancy	1.499 (1.111–2.023)	0.008	1.444 (1.022–2.041)	0.037^*^	1.406 (0.992–1.991)	0.055
Ongoing pregnancy	1.481 (1.105–1.984)	0.008	1.443 (1.031–2.020)	0.033	1.387 (0.988–1.948)	0.059
Biochemical miscarriage	0.331 (0.195–0.564)	<0.001	0.323 (0.174–0.599)	<0.001^*^	0.328 (0.176–0.611)	<0.001
Gestational diabetes	1.019 (0.570–1.819)	0.950	0.850 (0.428–1.688)	0.642	0.886 (0.446–1.762)	0.730
Gestational hypertension	0.767 (0.247–2.382)	0.765	0.652 (0.186–2.289)	0.505	0.662 (0.187–2.348)	0.523
Pre-eclampsia	0.717 (0.251–2.048)	0.580	0.732 (0.238–2.250)	0.586	0.749 (0.244–2.300)	0.613
Preterm delivery	1.066 (0.493–2.306)	0.870	1.273 (0.518–3.130)	0.599	1.274 (0.520–3.124)	0.596
Small for gestational age	0.729 (0.322–1.649)	0.447	0.712 (0.293–1.728)	0.452	0.700 (0.285–1.715)	0.435
Large for gestational age	0.997 (0.601–1.655)	0.992	1.064 (0.602–1.881)	0.832	1.084 (0.613–1.916)	0.782

*Modal 1 is adjusted for total testosterone, total gonadotropin dose (IU), oestradial level on HCG trigger day (pmol/L), progesterone level on HCG trigger day (nmol/L), No. of 2PN embryos, No. of cleavage embryos, No. of day-3 embryos suitable for transfer and endometrial thickness before transfer (cm). Modal 2 is adjusted for age, FSH, total testosterone, total gonadotropin dose (IU), oestradial level on HCG trigger day (pmol/L), progesterone level on HCG trigger day (nmol/L), No. of 2PN embryos, No. of cleavage embryos, No. of day-3 embryos suitable for transfer and endometrial thickness before transfer (cm). HRT cycle regimen was set to be the referent group*.

* *Value of Hosmer-Lemeshow Test goodness-of-fit <0.05*.

## Discussion

In ovulatory women with a good prognosis, we found that the incidence of maternal and neonatal complications was similar in natural cycle and HRT cycle regimens after frozen single blastocyst transfer. HRT FET was associated with increased risks of biochemical miscarriage in comparison to the natural FET. However, the natural cycle regimen did not result in a higher rate of live birth, clinical pregnancy or ongoing pregnancy than in the HRT cycle regimen after adjusting for potential confounders.

HRT cycle is also called hormone replacement cycle (HRC), programmed cycle (PC) or artificial cycle (AC) in different literatures (10, 19–21). For anovulatory women, exogenous hormone preparation is often preferred. In ovulatory women, artificial endometrial preparation may benefit from minimal monitoring and ease of scheduling transfers. However, the universal application of HRT cycles may have potential disadvantages including an increased cost, inconvenience and the potential adverse events (e.g., increased thrombotic risk) associated with estrogen supplementation (22). Natural cycle of endometrium preparation can only be offered to patients with an ovulatory cycle. Developing follicle and urine or serum luteinizing hormone levels are needed to be monitored. Detection of an LH surge, thawing and transfer can be planned accordingly. While ovulation is triggered by hCG administration, the dominate follicular formation could reduce cancellation rates, shortening the duration of monitoring and improving corpus luteum function. No statistically significant difference for live birth rate was noted between spontaneous and induced ovulation (13, 23). Groenewoud et al. summarizes the differences between the two regimens, no optimal minimal monitoring regimen in NC–FET has been determined, routine use of luteal phase support in NC–FET has not been shown to be advantageous but to increase treatment burden. Furthermore, the costs of both protocols were comparable (2).

In this multicenter clinical trial, local investigators decide endometrium preparation protocols and whether to use hCG for ovulation triggering according to their clinical routine. The baseline characteristics of total gonadotropin dose, E_2_ and progesterone level on day of trigger, the number of 2PN embryos, cleavage embryos and day-3 suitable in the natural cycles were different from the HRT cycles. Thus, it can be hypothesized that more follicular developed during IVF procedure in the HRT cycle regimen. Although hyperandrogenism have a higher risk of maternal and neonatal pregnancy complications (24, 25), total testosterone in both regimens was within the normal range, regardless of its effect on obstetric outcomes. In multivariate analysis, the effects of these differences were adjusted. We observed that endometrial thickness before transfer was significantly thicker in natural cycles compared with HRT cycles, and it is consistent with previous reports (9, 26). Natural cycles yielding an optimal endometrial thickness may compromise the window of implantation (WOI) (9).

Thus, optimal endometrial preparation and identification of the receptive window are important factors in the process of embryo implantation (27). Recent data from both simple histologic endometrial dating and transcriptomic microarray had shown that the window of implantation in hormonally prepared cycles for FET be delayed in about 25% women (28). Cluster analysis demonstrated that natural cycles were associated with a better endometrial receptivity transcriptome than HRT cycles (29).

As only a few high quality RCTs on the optimal preparation for FET are available (22), no substantial difference in live birth was obtained from a recent meta-analysis (30). Many studies did not conduct subgroup analysis of cleavage stage embryo and blastocyst transfer; no statistically significant difference for both clinical pregnancy and live birth were noted between natural cycles and HRT cycles (15, 19). In the patients with frozen cleavage embryo transfer, natural cycle and HRT cycle protocols yielded a comparable clinical pregnancy rate and live birth rate (10), while in frozen blastocyst transfer HRT cycles were associated with higher live birth rate (10, 31). Nonetheless, our study suggests that the live birth rate of frozen blastocyst transfer was better with the natural cycle preparation protocol before adjustment, supporting previous findings (9, 12). The increase of live birth rate in natural cycles may potentially be due to the lower risk of biochemical miscarriage. A prospective and observational cohort study by Cerrillo et al. (20) was performed and a higher miscarriage rate was observed in the hormone replacement cycles when compared to the natural cycles.

Many factors influence the live birth and clinical pregnancy rate following FET: female age and basal FSH level, the number of top-quality embryos and maximal endometrial thickness were the significant factors (9, 32). Therefore, when model 1 did not pass the Hosmer-Lemeshow test after adjusting the potential confounding factors, we added age and FSH to adjust. In NC-FET, the serum level of progesterone elevation present for 2 or more days before the LH surge (33), HCG trigger or not, and luteal support type may affect live birth rate (34). In HRT-FET, duration of both estrogen and progesterone supplementation and various routes of estrogen and progesterone administration are necessary to be considered (2). In our study, the disproportionate number of NC and HRT cycle was due to the discretion of local investigators, and this can lead to an inherent bias.

Numerous studies have tried to identify the optimal regimen of FET to obtain better pregnancy outcomes and to avoid or reduce adverse obstetric and neonatal outcomes. A large-scale registry-based study (7) on maternal and neonatal outcome of pregnancy after single embryo transfer showed that FET was associated with improved outcomes of preterm birth (PTB), low birth weight (LBW), and SGA compared with fresh transfer. Moreover, FET was associated with a statistically significant higher rate of placenta accreta and PIH. Compared to natural frozen cycles, higher rates of hypertensive disorders and preeclampsia in pregnancy were detected in HRT cycles (14, 15, 21). Recent studies focused on the number of corpus luteum (CL) (14, 35), which can impact obstetric outcomes. Programmed frozen embryo transfer without ovulation is absent of CL, and natural cycle FET due to spontaneous ovulation has a CL. There is an increased rate of preeclampsia in programmed FET cycles where no CL is present. The study showed highly increased rates of preeclampsia in HRT FET cycles compared to other FET protocols (14). Vascular health in early pregnancy was altered in women with aberrant numbers of CL (0 or >3) and might represent insufficient cardiovascular adaptation contributing to an increased risk of preeclampsia (35).

The average birth weight from HRT was significantly greater compared with NC (36). A higher risk of post-term delivery and Cesarean section were noted in patients who conceived singletons after HRT cycle compared with those who conceived after NC-FET (21, 36). FET singletons had an increased risk of being born LGA (8), but the frequencies of macrosomia were comparable between patients after NC-FET and HRT-FET (21). Artificial hormone circumstances in frozen embryo transfer during the HRT cycle may change the placental basal plate and be causatively associated with the amount of bleeding in deliveries (16). The research indicated the association of the thinned decidual layer with pregnancies after the frozen-thawed embryo transfer (16).

Although our study had a relatively large sample, it was neither designed nor powered to show differences in obstetric and neonatal complications. The strengths of this study included the multicenter source of the data, which enhanced the generalizability of our results. In addition, data collection on maternal and neonatal outcomes was obtained in a consistent way and adjustment was made for several confounders. There are also limitations to this study. First, though it was a relatively large-sample study from a randomized clinical trial, the selection of endometrial preparation regimen for frozen single blastocyst is not random. Our results need to be confirmed by future randomized controlled trials. Second, there were differences in baseline characteristics between the two protocols, which reduced comparability. Although multivariate analysis was performed, the interaction between the confounders was not clear. Additionally, only young women with a good prognosis were included, thus we should also be cautious to extend the results to patients with older age, poor ovarian response, or repeatedly previous failed IVF cycles whose risks of obstetric complications may be higher than good prognosis patients. Finally, elements of the pragmatic design such as whether to use HCG trigger in natural cycles, duration of both estrogen and progesterone supplementation, or choice of luteal support could have affected results. Thus, whether endometrium preparation with hormone replacement acts as an unfavorable factor for the occurrence of biomedical miscarriage warrants further study. If so, what the mechanism is also needs to be investigated.

## Conclusion

In summary, in women with ovulation there were no significant differences of maternal and neonatal complications in single frozen blastocyst transfer after natural cycle or HRT cycle regimens. Natural cycle was associated with a lower biomedical miscarriage rate.

## Data Availability Statement

All datasets generated for this study are included in the article/supplementary material.

## Ethics Statement

The studies involving human participants were reviewed and approved by Institutional Review Board of Center for Reproductive Medicine of Shandong University. The patients/participants provided their written informed consent to participate in this study.

## Author Contributions

YS was in charge of the trial conduct. JL, JZ, GH, JT, and YS designed the study. YS, JZ, GH, JT, ZW, and NX acquired the data. ZW, YP, and QJ performed the statistical analyses. YP, ZW, and QJ interpreted the data. JL wrote the first draft of the report with inputs from JZ, GH, and JT. YS provided comments, participated in additional discussions, and revised the paper. All authors approved the final version.

## Conflict of Interest

The authors declare that the research was conducted in the absence of any commercial or financial relationships that could be construed as a potential conflict of interest.
